# Prevalence of malnutrition inflammation complex syndrome among patients on maintenance haemodialysis at Muhimbili National Hospital in Tanzania: a cross-sectional study

**DOI:** 10.1186/s12882-020-02171-3

**Published:** 2020-11-30

**Authors:** Puneet K. Bramania, Paschal Ruggajo, Rimal Bramania, Muhiddin Mahmoud, Francis F. Furia

**Affiliations:** 1 School of Medicine, Muhimbili University of Health and Allied Sciences, Dar es Salaam, Tanzania; 2 Renal Unit, Muhimbili National Hospital, Dar es Salaam, Tanzania

**Keywords:** Malnutrition inflammation complex syndrome, Haemodialysis in Tanzania

## Abstract

**Background:**

Malnutrition, inflammation, and the combination thereof are predictors of poor outcomes in haemodialysis patients. Malnutrition Inflammation Complex Syndrome (MICS) is an accelerator of atherosclerosis and portends high mortality. Early recognition and treatment of MICS may help to improve the clinical outlook of such patients. This study investigated the prevalence of MICS and its associated factors among patients on maintenance haemodialysis at Muhimbili National Hospital (MNH) in Dar es Salaam, Tanzania.

**Methods:**

This was a prospective cross-sectional observational study done among 160 adult patients on maintenance haemodialysis at MNH in 2019. All participants provided written informed consent. Questionnaires were used to collect data and patients’ blood was tested for complete blood count (CBC), C-reactive protein (CRP), ferritin, transferrin, creatinine, urea, total cholesterol, and albumin. The Malnutrition Inflammation Score was used to assess MICS and its severity. Data analysis was done using the SPSS 20 software.

**Results:**

Of the 160 patients included in the study, 111 (69.4%) were male. The mean age (±SD) of patients and mean duration (±SD) on haemodialysis were 52.2(13.3) years and 22(18) months respectively. MICS was prevalent in 46.3% (mild in 24.4% and moderate to severe in 21.9%).

Long-term haemodialysis (> 4 years) was an independent predictor of MICS [Adjusted Odds Ratio, AOR 5.04 (95% CI: 1.33–19.2), *p* < 0.05]. Hypercholesterolaemia was a negative predictor of MICS [AOR 0.11 (95% CI: 0.01–0.97), *p* < 0.05]. Patients with MICS had significantly lower mean body mass index, serum albumin, total cholesterol, transferrin, haemoglobin, and creatinine levels. The presence of MICS was higher in underweight patients and those who had inflammation. Haemodialysis adequacy did not correlate with MICS.

**Conclusion:**

Malnutrition Inflammation Complex Syndrome is relatively common among patients on haemodialysis in Dar es Salaam, Tanzania. Our study has shown a longer duration on haemodialysis to be associated with the occurrence of MICS; on the contrary, having hypercholesterolaemia seems to be protective against MICS consistent with the concept of reverse epidemiology. Patients on haemodialysis should be assessed regularly for malnutrition and inflammation and should receive appropriate and timely treatment to reduce the burden of associated morbidity, and mortality to these patients.

## Background

Chronic kidney disease (CKD) is associated with high morbidity and mortality [1]. The community prevalence of CKD in a study done in Northern Tanzania has been estimated to be 7%. The urban prevalence is higher than in rural (15.2% versus 2.0%) [2]. Haemodialysis (HD) services provision in Tanzania have been continually rising over the past decade from one haemodialysis center to 27 centers in 2018, most of these are in large cities [3]. There were 933 patients receiving HD in the country as of 2018 [3]. Haemodialysis services in Tanzania are largely reimbursed by the National Health Insurance Fund (NHIF), others pay ‘out of pocket’ and for a few patients HD services are covered by other private insurance or companies. Those paying ‘out of pocket’ may not be able to afford HD services and have a higher risk of poor outcomes [4]. This can be attributed to non-adherence to the standard three times per week haemodialysis. A recent multi-center study by Somji et al. in Dar es Salaam, Tanzania reported a high proportion of inadequate HD (59.4%), however, it did not find a significant correlation between HD adequacy and the body mass index (BMI). In the same study, 9.8% of the HD patients were underweight [5].

Malnutrition and inflammation are common problems in patients on HD and are associated with adverse outcomes [6–8]. The combination of these two entities termed Malnutrition Inflammation Complex Syndrome (MICS) portends higher mortality among patients on HD [7, 9]. High prevalence rates for MICS have been reported among patients on HD, a study conducted in Brazil among HD patients of African descent reported MICS in 42.4% while Matiko reported a prevalence of 61.2% among HD patients in Kenya [10, 11].

Malnutrition among HD patients is attributed to several factors including limited dietary intake, dialysis-related nutrient loss, oxidative stress, metabolic acidosis, hyper-metabolism, and chronic inflammation-induced protein breakdown [8, 12]. HD adequacy has been found to be correlated with inflammatory markers like CRP [13]. The prevalence of chronic inflammation among patients on HD ranges from 30 to 50%, this is principally attributed to the uremic milieu that induces pro-inflammatory cytokines, which have negative effects on protein synthesis and may accelerate atherosclerosis [8, 14]. The interplay between atherogenic effects from persistent inflammation and malnutrition contributes to the Malnutrition-Inflammation-Atherosclerosis (MIA) syndrome which has high mortality [6, 15, 16]. MICS may serve as a proxy indicator of atherosclerosis in these patients and its recognition may be important in guiding the treatment of underlying cardiovascular disease [17].

Malnutrition in CKD leads to increased morbidity thus its early recognition and treatment have the potential of reducing the cost of health care. Tanzania has a high burden of CKD; however, there is scarce data on the magnitude of malnutrition and inflammation among these patients including those receiving HD therapy. This study aimed to explore the magnitude of MICS among patients on maintenance haemodialysis and its associated factors at MNH in Dar es Salaam, Tanzania.

## Methods

### Study design, duration, and setting

It is a multi-center (two HD centers of MNH) prospective cross-sectional observational study. The study was conducted between September and November 2019 among patients undergoing haemodialysis at the two HD centers of Muhimbili National Hospital (Upanga and Mloganzila) in Dar es Salaam, Tanzania.

### Study population

All adult patients receiving maintenance HD therapy at the two dialysis units of MNH for at least three months were eligible. Patients who were mentally incapacitated, very sick, bedridden patients, patients with altered levels of consciousness, and those in respiratory distress were excluded.

### Sample size calculation

The sample size for this study was estimated based on the prevalence of MICS of 61.2% reported by Matiko among HD patients in Kenya, using the following formula below [18].

$$ \mathrm{n}=\frac{{\mathrm{n}}_{\mathrm{o}}\;\mathrm{x}\;\mathrm{N}}{{\mathrm{n}}_{\mathrm{o}}+\left(\mathrm{N}-1\right)} $$ where, $$ {\mathrm{n}}_{\mathrm{o}}=\frac{{\mathrm{Z}}^2\;\mathrm{p}\left(1-\mathrm{p}\right)}{{\mathrm{e}}^2} $$

Whereby:

n = Minimum sample size needed.

n_o_ = Is the sample size without considering the finite population correction factor.

N = The total number of haemodialysis patients at both renal centers of MNH (At the time of preparation of the study protocol there were 284 patients on HD).

Z = Standard normal deviation = 1.96 (at 95% Confidence Interval).

p = Prevalence of MICS. *p* = 61.2% = 0.612 among HD patients in a study done in Kenya [11].

e = Margin of error which was set at 5% = 0.05.

Substituting these figures in the formula gives; *n* = 160.

Therefore, the minimum required sample size was 160 patients.

### Sampling method

Simple random sampling was utilized to select participants for this study. A list of all patients on HD therapy was obtained from the HD units record books at the beginning of the study. Patients were assigned numbers then the Stat Trek’s Random Number Generator was used to select participants recruited in this study [19].

### Data collection methods

Data were actively collected by an ad hoc questionnaire (Supplementary S1) which included participant’s demographic and clinical data, questions about dietary intake, gastrointestinal symptoms; functional capacity, and co-morbid status. These were taken from the Malnutrition Inflammation Score (MIS) [7]. The Malnutrition Inflammation Score is a comprehensive assessment of the nutritional and inflammation status of patients. It has 10 components (5 from medical history, 3 from physical examination, and 2 from laboratory parameters: albumin and transferrin) each has a severity ranging from 0 (normal) to 3 (severely abnormal). The total of all 10 components ranges from 0 (normal) to 30 (severely malnourished); higher scores imply a more severe degree of malnutrition and inflammation. The MIS components include (i) Change in end dialysis dry weight over past 3 months (ii) Dietary intake (iii) Gastrointestinal symptoms (iv) Functional capacity (v) Co-morbidity including the number of years on dialysis (vi) Loss of fat stores (vii) Signs of muscle wasting (viii) Body mass index (BMI) (ix) Serum albumin and (x) Serum Total Iron binding capacity (TIBC) or serum transferrin. Dietary intake was scored 0 for the usual intake of solid foods, with no recent decrease in the amount/quality of meals. A slightly suboptimal solid diet was scored 1, a full-liquid diet or moderate decrease in dietary intake was scored 2 and a score of 3 indicated a hypocaloric liquid diet or starvation. The full description and grading of the severity of these components is available in the study by Kalantar-Zadeh et al., who is the inventor of this scoring system [7].

Body (or somatic) mass was assessed using the body mass index (BMI) that was determined using participants’ post-dialysis dry weight and height. BMI was computed using the weight (kg) and height (m) and was expressed in kg/m^2^: BMI = Weight (kg) ÷ [Height (m)] ^2^.

Body weight was measured using a standard weighing scale while the patient had not worn shoes and a stadiometer was used to measure the height of participants to the nearest centimeter. For patients who were unable to stand in an upright position on the stadiometer, the length from top of the head to the plantar surface of the foot while lying supine was taken in lieu of their height.

Patients’ dry weight in the preceding 3 months was obtained from their HD records and were compared with current post-dialysis dry weight to calculate the percentage change. The loss of subcutaneous fat was assessed below the eyes, triceps, biceps, and chest. Signs of muscle wasting were assessed at the temple, clavicle, scapula, ribs, quadriceps, knee, and interosseous regions. Subcutaneous fat loss, muscle wasting, and BMI were categorized based on the MIS [7].

### Laboratory tests

Blood specimen (10 mls) was obtained from each participant before and after dialysis and was sent to the Central Pathology Laboratory at MNH. Laboratory testing was performed to determine complete blood count (CBC), C-reactive protein (CRP), ferritin, transferrin, creatinine, urea, total cholesterol, and albumin. Post-dialysis creatinine and urea were also determined. CELL DYN 3700 and ARCHITECT PLUS machines were used to analyze complete blood count and serum biochemistry respectively. CRP was analyzed at the Muhimbili University of Health and Allied Sciences (MUHAS) Clinical Research Laboratory using the COBAS INTEGRA 400, which uses Finecare CRP Rapid Quantitative test that is based on the fluorescence immunoassay.

### Data management and analysis

Questionnaires were checked for completeness after which data were entered into the Statistical Package for Social Sciences (SPSS) version 20 for data cleaning and analysis. Data were summarized into frequency distribution and two-way tables. Association between categorical variables was determined using the Chi-square and Fischer’s exact tests while continuous variables association was determined using student t-test and analysis of variance (ANOVA). Non-parametric variables were compared using Mann-Whitney U-test. Binary Logistic regression analysis (univariate and multivariate) was performed to determine the predictors/factors associated with MICS. A two-tailed *p*-value of less than 0.05 was regarded as statistically significant and the Hosmer-Lemeshow test was used to assess goodness of fit for the final logistic regression model.

### Study variables

The main outcome variable was the presence of MICS as defined by an aggregate MIS of 6 or more. The severity of MICS was graded as: A MIS of less than 6 was categorized as *Normal*, between 6 and 10 as *Mild* and a score of 11 or more was considered as *Moderate to Severe* [11]. Body size (BMI) categories for this study were defined as *Underweight* (BMI < 18.5 kg/m^2^), *Well-nourished* (BMI 18.5 to 24.99 kg/m^2^) and *Overweight or Obese* (BMI > 25 kg/m^2^) [20]. A pre-dialysis CRP level above 5 mg/l was defined as having *Inflammation* [21]. Anemia was defined by a haemoglobin level below 13 g/dl in males and below 12 g/dl for females based on KDIGO guidelines [22]. Hypercholesterolaemia was defined by a serum total cholesterol (TC) level above 220 mg/dl (the upper limit of the normal range at MNH Laboratory). *Adequate dialysis* was defined as having Urea Reduction Ratio (URR) of 65% or more (calculated as URR = (Pre-dialysis Urea - Post-dialysis Urea)/Pre-dialysis Urea X 100%) [23].

## Results

**Fig. 1:**
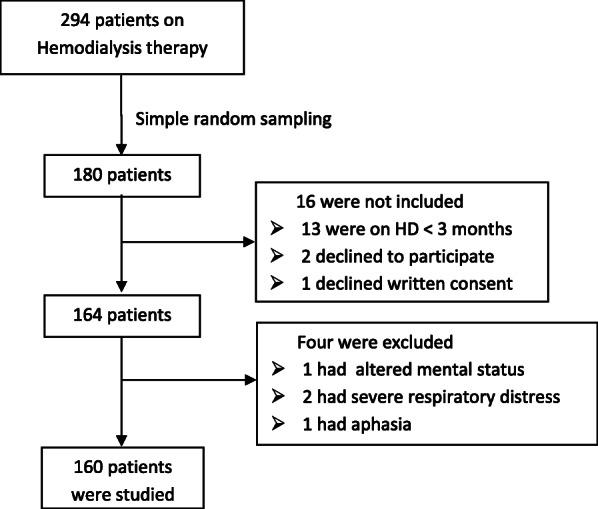
Flow diagram to show recruitment of participants

Among the 294 patients on HD therapy in both centers at the beginning of the data collection, 160 participants were studied/analyzed. (Fig. 1).

### Baseline socio-demographic-clinical characteristics of participants

As planned, 160 patients were included in the evaluation. 17.1% of the males and 32.7% of the females resulted in moderate/severe MICS status. The mean age (±SD) of patients was 52.2(13.3) years and the mean duration on HD therapy was 22 (+ 18) months (range: 3 to 126 months). Most of them, 132 (82.5%) were receiving three times per week HD therapy and 76 (47.5%) had an arteriovenous fistula (AVF) as the vascular access in use for HD. The majority of them, 136 (85%) were living with a partner, and 144 (90%) were covered by health insurance. Comorbidity-wise, almost all 154 (96.3%) had hypertension that was as well the commonest (43.1%) reported cause of End-Stage Renal Disease (ESRD). Diabetes mellitus was prevalent in 43.1% and anemia was present in 153 (95.6%) patients. (Table 1).

**Table 1:** Socio-demographic and clinical characteristics of patients in relation to severity of MICS (*N* = 160)

Characteristics	n (%)	MICS status			***p***-value
		No	Mild	Mod-Severe	
**Age groups (years)**					
18–39	30 (18.8%)	12 (40.0%)	9 (30.0%)	9 (30.0%)	0.310^a^
40–59	75 (46.9%)	46 (61.3%)	17 (22.7%)	12 (16.0%)	
> 60	55 (34.4%)	28 (50.9%)	13 (23.6%)	14 (25.5%)	
**Mean Age** (+ SD) years	52.2 + 13.3	53.2 + 11.8	49.7 + 14.4	52.6 + 15.3	0.397^c^
**Gender**					
Male	111 (69.4%)	65 (58.6%)	27 (24.3%)	19 (17.1%)	0.072^a^
Female	49 (30.6%)	21 (42.9%)	12 (24.5%)	16 (32.7%)	
**Marital Status**					
Living with a partner	136 (85.0%)	74 (54.4%)	32 (23.5%)	30 (22.1%)	0.837^a^
Single	24 (15.0%)	12 (50.0%)	7 (29.2%)	5 (20.8%)	
**Level of Education**					
No Formal / Primary	41 (25.6%)	19 (46.3%)	13 (31.7%)	9 (22.0%)	0.429^a^
Post-primary	119 (74.4%)	67 (56.3%)	26 (21.8%)	26 (21.8%)	
**Mode of Payment**					
Health Insurance	144 (90.0%)	78 (54.2%)	34 (23.6%)	32 (22.2%)	0.776^b^
Out Of Pocket	16 (10.0%)	8 (50.0%)	5 (31.2%)	3 (18.8%)	
**Frequency of HD**					
Thrice/week	132 (82.5%)	73 (55.3%)	30 (22.7%)	29 (22.0%)	0.575^a^
Twice/week	28 (17.5%)	13 (46.4%)	9 (32.1%)	6 (21.4%)	
**Duration of HD (years)**					
< 1	54 (33.8%)	34 (63.0%)	16 (29.6%)	4 (7.4%)	< 0.001^b^
1–4	92 (57.4%)	49 (53.3%)	22 (23.9%)	21 (22.8%)	
> 4	14 (8.8%)	3 (21.4%)	1 (7.1%)	10 (71.4%)	
**Duration on HD (months)**					
Mean (+ SD)	22 + 18	17.9 + 12.3	18 + 13.5	34.7 + 26.3	< 0.001^c^
**Vascular Access**					
AV Fistula	76 (47.5%)	42 (55.3%)	16 (21.1%)	18 (23.7%)	0.644^a^
Central Venous Catheter	84 (52.5%)	44 (52.4%)	23 (27.4%)	17 (20.2%)	
**Dialysis Adequacy**					
Inadequate (URR < 65%)	38 (23.8%)	19 (50.0%)	8 (21.1%)	11 (28.9%)	0.479^a^
Adequate (URR > 65%)	122 (76.3%)	67 (54.9%)	31 (25.4%)	24 (19.7%)	
**Diabetes Mellitus**					
Yes	69 (43.1%)	40 (58.0%)	12 (17.4%)	17 (24.6%)	0.198^a^
No	91 (56.9%)	46 (50.5%)	27 (29.7%)	18 (19.8%)	
**Hypertension**					
Yes	154 (96.3%)	83 (53.9%)	37 (24.0%)	34 (22.1%)	0.863^b^
No	6 (3.7%)	3 (50.0%)	2 (33.3%)	1 (16.7%)	
**Hypercholesterolaemia**					
Yes (TC > 220 mg/dl)	9 (5.6%)	8 (88.9%)	1 (11.1%)	0 (0.0%)	0.133^b^
No (TC < 220 mg/dl)	151 (94.4%)	78 (51.7%)	38 (25.2%)	35 (23.2%)	
**HIV Infection**					
Yes	15 (9.4%)	5 (33.3%)	5 (33.3%)	5 (33.3%)	0.223^b^
No	145 (90.6%)	81 (55.9%)	34 (23.4%)	30 (20.7%)	
**HBV Infection**					
Yes	9 (5.6%)	5 (55.6%)	3 (33.3%)	1 (11.1%)	0.740^b^
No	151 (94.4%)	81 (53.6%)	36 (23.8%)	34 (22.5%)	

^a^ Chi-square test, ^b^ Fisher’s exact test, ^c^ ANOVA

### Prevalence and severity of malnutrition inflammation complex syndrome (MICS)

**Fig. 2:**
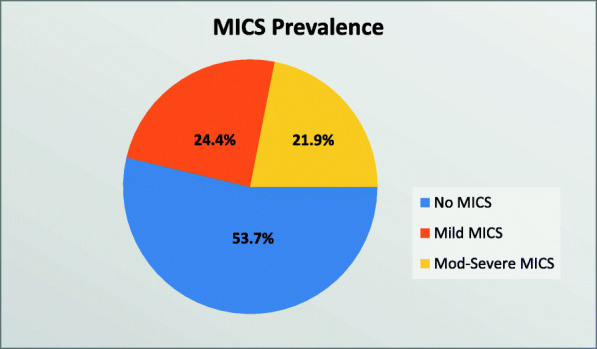
Pie chart to show prevalence of MICS among patients on maintenance haemodialysis at MNH

Of the 160 patients on maintenance haemodialysis, 74 (46.3%) had MICS and these were categorized as mild MICS in 39 (24.4%) and moderate to severe MICS in 35 (21.9%) of the patients. (Fig. 2).

### Malnutrition inflammation score and its parameters

The Malnutrition-Inflammation-Score (MIS) ranged from 0 to 28 with a median MIS of 5 and a mean (±SD) MIS of 7.6 (±5.1). Compared to well-nourished patients, the mean MIS was significantly higher among underweight patients (14.8 vs 6.2, *p* < 0.001). Overall dry weight loss > 0.5 kg over the preceding 3 months was present in 104 (65%) patients and severe dry weight loss (> 5%) was present in only 13 (8.1%) patients. Dietary intake was good in almost half; 87 (54.4%) patients, and severe dietary limitations were present in only four (2.5%) patients. About one-third of the patients, 50 (31.2%) had some gastrointestinal symptoms. Physical examination revealed 98 (61.3%) patients had normal fat stores and 83 (51.9%) patients had no muscle wasting. Severe muscle wasting was present in 14 (8.8%) patients.

### Body size, inflammation and its association with MICS

Among the 160 patients, 27 (16.9%) were underweight and among the 103 patients whose CRP was measured, 84 (81.6%) had inflammation (CRP > 5 mg/l). The presence of MICS was higher in underweight patients. Also, inflammation was significantly associated with MICS. (Table 2).

**Table 2:** Association between Body size and Inflammation with MICS

Body size (N = 160)	MICS Present	MICS Absent	TOTAL	***p***-value
Underweight	26 (96.3%)	1 (3.7%)	27 (100%)	< 0.001^a^
Normal weight	46 (48.4%)	49 (51.6%)	95 (100%)	
Over-weight/Obese	2 (5.3%)	36 (94.7%)	38 (100%)	
**Inflammation** (*N* = 103)				
Present	53 (63.1%)	31 (36.9%)	84 (100%)	< 0.001^a^
Absent	2 (10.5%)	17 (89.5%)	19 (100%)	

^a^Test Statistic: Fisher’s exact test

### Factors associated with malnutrition inflammation complex syndrome

In univariate analysis female gender, longer duration on HD, hypercholesterolaemia, and having HIV associated with the occurrence of MICS. For multivariate analysis, the purposive selection of variables showing a univariate *p* < 0.2 were included in the final regression logistic model. Patients with high cholesterol levels seemed to be protected against the development of MICS. Finally, patients longer on haemodialysis significantly associated with a higher risk for MICS. (Table 3).

**Table 3:** Factors associated with Malnutrition Inflammation Complex Syndrome (N = 160)

Characteristics	COR^a^ (95% CI)	*p*-value	AOR^b^ (95% CI)	*p*-value
Age groups				
< 60 years	Ref		–	–
> 60 years	1.19 (0.62–2.3)	0.60		
Gender				
Male	Ref		Ref	
Female	1.88 (0.95–3.72)	0.07	2.03 (0.97–4.25)	0.061
Marital Status				
Living with a partner	Ref		–	–
Single	1.19 (0.5–2.84)	0.69		
Mode of payment				
Health Insurance	Ref		–	–
Paying out of pocket	1.18 (0.42–3.32)	0.75		
Duration on HD				
< 4 years	Ref		Ref	
> 4 years	4.83 (1.29–18)	0.02	5.04 (1.33–19.2)	0.018
Frequency of HD				
Thrice/week	Ref		–	–
Twice/week	1.43 (0.63–3.24)	0.39		
Vascular Access				
AV Fistula	Ref		–	–
Central Venous Catheter	1.12 (0.6–2.09)	0.72		
Dialysis Adequacy				
Inadequate (URR < 65%)	1.22 (0.59–2.53)		–	–
Adequate (URR > 65%)	Ref	0.60		
Diabetes Mellitus				
Yes	0.74 (0.4–1.4)		–	–
No	Ref	0.35		
Hypertension				
Yes	0.86 (0.17–4.37)		–	–
No	Ref	0.85		
Hypercholesterolaemia				
Yes (TC > 220 mg/dl)	0.13 (0.02–1.1)		0.11 (0.01–0.97)	0.047
No (TC < 220 mg/dl)	Ref	0.06	Ref	
HIV Infection				
Yes	2.53 (0.82–7.78)		2.55 (0.78–8.4)	0.123
No	Ref	0.11	Ref	
HBV Infection				
Yes	0.93 (0.24–3.58)		–	–
No	Ref	0.91		

Hosmer Lemeshow Test for Multivariate Logistic regression, *p* = 0.656 → Goodness of fit present

^a^Crude Odds Ratio (COR) ^b^ Adjusted Odds Ratio (AOR)

### Association between MICS and nutritional, inflammatory, and other laboratory parameters

Patients with MICS had significantly lower mean dry-weight, BMI, haemoglobin, albumin, total cholesterol, transferrin, and creatinine levels. The median CRP level was significantly higher in patients with MICS. (Table 4).

**Table 4:** Association between MICS and nutritional, inflammatory and other laboratory parameters

Parameters	N	MICS Present (Mean **+** SD)	MICS Absent (Mean **+** SD)	***p***-value
**Nutritional parameters**				
Dry weight (kg)	160	56.6 + 9.4	68.9 + 10.9	< 0.01 ^a^
BMI (kg/m^2^)	160	20.2 + 2.7	24.4 + 3.8	< 0.01 ^a^
Albumin (g/dl)	160	3.47 + 0.49	3.94 + 0.36	< 0.01 ^a^
Total Cholesterol (mg/dl)	160	138.7 + 34.4	163.3 + 40.6	< 0.01 ^a^
**Inflammatory markers**				
Transferrin (mg/dl)	160	164.1 + 40.2	217.8 + 44.4	< 0.01 ^a^
CRP (mg/l)	103	22.5 (IQR = 12.7–43.3)	10.45 (IQR = 5.0–16.8)	< 0.01^b^
Ferritin (ng/ml)	102	138.2 (IQR = 75–349.8)	100.2 (IQR = 49.5–216.7)	NS ^b^
**Other laboratory parameters**				
WBC (× 10^9^/L)	160	5.21 + 2.78	4.93 + 1.79	NS ^a^
Haemoglobin (g/dl)	160	8.8 + 2.0	9.7 + 1.8	< 0.01 ^a^
Pre-dialysis Cr (μmol/l)	160	768 + 316	911 + 412	0.016 ^a^
Post-dialysis Cr (μmol/l)	160	267 + 128	361 + 202	< 0.01 ^a^
Urea Reduction Ratio (URR)%	160	69.9 + 13.6	70.4 + 9.0	NS ^a^

*NS* Not significant (*p* > 0.05), *IQR* Interquartile Range

^a^ Test Statistic: Student’s t-test, ^b^ Test Statistic: Mann-Whitney U test

## Discussion

In this cross-sectional study, 160 haemodialysis patients were recruited to determine the prevalence of Malnutrition Inflammation Complex Syndrome and its associated factors at MNH in Dar es Salaam, Tanzania. More than two-thirds of the patients, 111 (69.4%) were male and the majority 132 (82.5%) were on HD three times per week (as per KDOQI recommendations). The prevalence of MICS was 46.3% and based on severity; MICS was categorized as mild in 24.4% and moderate to severe in 21.9% of the patients. In multivariate analysis, longer duration on HD (> 4 years) was a significant predictor of MICS and hypercholesterolaemia was a negative predictor of MICS. Patients with MICS had significantly lower mean BMI, albumin, total cholesterol, transferrin, haemoglobin, and creatinine levels.

The prevalence of MICS found in this study may not reliably reflect the situation in Tanzania due to rural-urban disparities in access to dialysis services [3]. A lower prevalence of MICS was noted in this study as compared to 61.2% reported by Matiko in a study conducted among HD patients in Kenya [11]. This difference could be attributed to the adequacy of dialysis as indicated by lower frequency (two times weekly) of dialysis in the study by Matiko as compared to our study in which most patients were getting three times per week dialysis [11].. Inadequate dialysis can lead to persistently high urea levels, subsequent inflammation, and protein catabolism [12]. However, in this study, HD adequacy as assessed by URR did not associate with MICS. In this study, moderate to severe MICS was noted in 21.9% of HD patients, this is similar to a study from South Africa that reported it to be 22% [24].

The prevalence of underweight HD patients was 16.9%; this was higher than the prevalence of 9.8% reported by Somji et al. in a study conducted in Dar es Salaam, Tanzania among HD patients [5]. The prevalence of underweight HD patients found in our study was lower when compared to other studies in African countries; 20.9% in Kenya [11], 29.2% in Niger [25], and 28.3% in Cameroon [26]. Patients with MICS have varying degrees of muscle wasting [12, 17]. In this study, patients with MICS had a mean BMI of 20.2 + 2.7 kg/m^2^ and almost half (48.4%) of the patients with normal BMI had MICS, this implies that BMI alone is not accurate for nutritional assessment in HD patients. The Malnutrition Inflammation Score is a better tool in the assessment of the nutritional status of HD patients and has been found to better correlate with morbidity, quality of life, and mortality [7, 9, 10]. Several factors affect the nutritional status of HD patients; decreased calorie intake, inadequate dialysis, metabolic acidosis, persistent inflammation, and HD-related catabolism [8, 12]. In this study, almost half of the patients had reported some deterioration in dietary intake and almost a third of patients reported gastrointestinal symptoms that may limit adequate dietary intake.

In our study, inflammation was present in 81.6% of the patients; this was lower than that found in the Kenyan study where all patients had raised CRP [11]. Inflammation in HD patients is common and largely attributed to persistent uremia. There are multiple other triggers including HD procedure itself, dialysate quality, and bio-incompatibility of dialysis membranes [4, 9]. The median CRP in our study was significantly higher in patients with MICS, this is consistent with a study done in Mexico [27].

In this study, patients on HD for a longer duration (> 4 years) had 5 times the odds of having MICS compared to those on HD for less than 4 years. This finding is consistent with findings from a study by Omari et al. who reported a long duration of dialysis to be linked with malnutrition inflammatory syndrome in Palestine [28]. The longer duration on HD therapy results in prolonged exposure of patients to negative effects of HD including dialysis-related inflammation, loss of nutrients, and increased energy expenditure that subsequently can result in malnutrition [12]. This highlights the need for efforts to increase uptake of HD patients into the kidney transplantation program to limit the time spent on chronic HD therapy.

Low socioeconomic status and limited access to health insurance significantly influence the adequacy of haemodialysis [4]. We found that the small group of patients who were paying ‘out of pocket’ (ie. not insured) had slightly higher odds of having MICS. There was a significant difference in the frequency of HD among these two groups; 87.5% of those paying ‘out of pocket’ versus 9.7% of those who had health insurance were having twice per week HD (*p* < 0.05). Thus health insurance offers the best option for sustaining the adherence to standard long-term haemodialysis.

Aging has been reported to correlate with MICS but did not show this effect in our study. This might be attributed to the younger age of patients in this study compared to that reported by Rambod et al. in a study among HD patients in California who found a higher burden of MICS among older patients on HD [9]. Similarly, other studies on MICS done in Kenya, Brazil, and Mexico also had a younger population; mean age < 50 years [10, 11, 27]. Some of the possible reasons could be due to variations in the profile of the HD patients which can be affected by differences in survival, recruitment in renal transplantation, and the access to sustainable maintenance HD services. MICS among older patients is attributed largely to co-morbidities including depression that may also affect the dietary intake of these patients.

In this study, the mean total cholesterol level was significantly lower in patients with MICS, which is contrary to a report by Valencia et al. that reported no difference in cholesterol levels between dialysis patients with and without MICS in Mexico [27]. Cholesterol is an important nutritional biomarker among patients on HD therapy and hypercholesterolaemia may be protective in these patients unlike the general population [29, 30].

Serum albumin was noted to be significantly lower among patients with MICS; this was an expected finding consistent with reports from other similar studies [9, 10]. Albumin is a good indicator of protein-energy malnutrition in CKD. Hypoalbuminaemia is attributed to visceral protein loss among patients undergoing HD as well as peritoneal dialysis [12]. Serum creatinine levels have also been reported to be lower among patients with MICS [10, 27].

The phenomenon of hypocholesterolaemia and hypocreatininaemia among patients on chronic dialysis, accompanied by MICS is referred to as ‘*reverse epidemiology*’. Reverse epidemiology is paradoxically associated with poor cardiovascular outcomes [29, 30], therefore health care providers providing chronic dialysis therapy should be vigilant and monitor these markers regularly to improve outcomes of chronic dialysis.

### Strengths and limitations

This is the first study in Tanzania to explore the burden of malnutrition and inflammation in HD patients. The other strength of this study was that it also assessed the relationship between nutritional and inflammatory biomarkers with MICS and was shown to be correlated hence reinforces the need for synergistic assessment of these parameters.

In view of the study settings, the data may not be generalized for the whole country. Another limitation of this study was the observer bias while measuring fat and muscle loss. Systematic errors during the measurement of anthropometric indices could have occurred. There might be some selection bias from (i) larger number of health insured patients compared to the small number of patients paying ‘out of pocket’ (ii) exclusion of a small group of very sick and mentally debilitated patients. This may underestimate the outcome. The CRP test was not done for all patients; this was due to financial restrictions and other logistic challenges.

## Conclusion

Malnutrition Inflammation Complex Syndrome (MICS) is prevalent among haemodialysis patients at MNH with a significantly higher burden among those with a long duration of haemodialysis therapy. Hypercholesterolaemia was noted to be protective against the occurrence of MICS consistent with the concept of reverse epidemiology. Hypoalbuminaemia and hypocholesterolemia in haemodialysis patients should prompt further investigations for their nutritional and inflammation status.

We recommend that patients on maintenance haemodialysis should be assessed regularly and treated accordingly for malnutrition and inflammation with more emphasis on patients longer on haemodialysis. We also recommend further studies at our set up to explore the possible causes of the high prevalence of MICS.

## Supplementary Information

**Additional file 1: Supplementary S1**. Questionnaire on MICS.

## Data Availability

The dataset generated and/or analyzed during the current study is available from the corresponding author on reasonable request.

## Abbreviations

ANOVA: Analysis of variance
AOR: Adjusted Odds Ratio
AVF: Arteriovenous fistula
BMI: Body Mass Index
CI: Confidence Interval
CKD: Chronic Kidney Disease
COR: Crude Odds Ratio
CRP: C-reactive protein
ESRD: End Stage Renal Disease
HD: Haemodialysis
HBV: Hepatitis B Virus
HIV: Human Immunodeficiency Virus
IQR: Interquartile Range
KDOQI: Kidney Disease Outcome Quality Initiative
MIA: Malnutrition Inflammation Atherosclerosis syndrome
MICS: Malnutrition Inflammation Complex Syndrome
MIS: Malnutrition Inflammation Score
MNH: Muhimbili National Hospital
MUHAS: Muhimbili University of Health and Allied Sciences
NHIF: National Health Insurance Fund
TC: Total Cholesterol
URR: Urea Reduction Ratio
WBC: White cell count
