# Supplementary material for: Prevalence of malnutrition inflammation complex syndrome among patients on maintenance haemodialysis at Muhimbili National Hospital in Tanzania: a cross-sectional study
Source: BMC Nephrol. 2020 Nov 30;21:521. doi: 10.1186/s12882-020-02171-3 (PMC7708158; doi:10.1186/s12882-020-02171-3)
Supplement: Supplementary file 1 — Additional file 1: Supplementary S1. Questionnaire on MICS. [file 12882_2020_2171_MOESM1_ESM.docx]

**MUHIMBILI UNIVERSITY OF HEALTH AND ALLIED SCIENCES SN: ……..**

**QUESTIONNAIRE ON MALNUTRITION INFLAMMATION COMPLEX SYNDROME (MICS) AMONG PATIENTS ON MAINTENANCE HEMODIALYSIS AT MUHIMBILI NATIONAL HOSPITAL**

The aim of this study is to determine the prevalence of Malnutrition Inflammation Complex Syndrome among patients on maintenance hemodialysis and its associated factors at Muhimbili National Hospital (MNH) in Dar-es-Salaam, Tanzania.

**Instructions:**

1. This questionnaire consists of three (3) sections: A, B and C

These sections involve questions that you will be asked and the researcher will fill the response in the appropriate space. It will also involve some physical examination and measurements.

2. Circle the response where appropriate or put a ‘’ √ ‘’ mark where applicable.

3. Fill in the response in space provided for some questions and measurements.

**SECTION A: Patient particulars and clinical information**

1. Gender: (i) Male (ii) Female

2. What is your year of birth? …………….. Age ………… years

(i) 18-39 years (ii) 40-59 years (iii) > 60 years

3. For how long have you been on hemodialysis therapy? Duration …………. months

(i) < 1 year (ii) 1-4 years (iii) > 4 years

1. How frequently do you have hemodialysis therapy per week?

(i) Thrice (ii) Twice (iii) Once or intermittent

1. What is your Current Vascular Access for Hemodialysis?

(i) Arteriovenous fistula (AVF) (ii) Central venous catheter (iii) AV graft

6. What is your marital status?

(i) Married (ii) Divorced (iii) Widow/widower (iv) Single (v) Cohabiting

7. What is your Highest-level level of education attained?

(i) No formal education (ii) Primary (iii) Secondary (iv) Higher

8. What is your Occupation Status?

(i) Retired (ii) Stopped working (ii) Currently working (iii) Not working (No job/student)

What is your Occupation? ………………………………………………………………..

9. How do you pay for the hemodialysis services?

(i) Health Insurance (ii) Paying out of pocket

10. Do you have any of these illnesses? Put a ’√’ mark where applicable

| Diabetes Mellitus |  | Hyperlipidemia |  | Hepatitis B Infection |  |
| --- | --- | --- | --- | --- | --- |
| Hypertension |  | HIV Infection |  | Hepatitis C Infection |  |

Other Co-morbidities: ……………………………………………………………………

11. What was the cause of your Chronic Renal Failure? Circle ONE

(i) DM (ii) HTN (iii) DM/HTN (iv) HIV (v) Unknown (vi) Other:…………………….

**SECTION B: Medical history components of Malnutrition Inflammation Score (MIS)**

| **Patient related medical history** (Circle one value that apply)   \| **Dietary intake** \| \| \| \| \| --- \| --- \| --- \| --- \| \| 0  Good appetite and no deterioration of diet intake \| 1  Somewhat sub-optimal solid diet intake \| 2  Moderate overall decrease to full liquid diet \| 3  Hypo-caloric liquid  to starvation \| \| **Gastrointestinal (GI) symptoms** \| \| \| \| \| 0  No symptoms with good appetite \| 1  Mild symptoms, poor appetite or nauseated occasionally \| 2  Occasional vomiting or moderate GI symptoms \| 3  Frequent diarrhea or  vomiting/severe anorexia \| \| **Functional Capacity Assessment (nutritionally related functional impairment)** \| \| \| \| \| 0  Normal to improved functional capacity, feeling fine \| 1  Occasional difficulty with baseline ambulation, or feeling tired frequently \| 2  Difficulty with otherwise independent activities (eg. going to the bathroom) \| 3  Bed/chair-ridden, or  little to no physical  activity \| \| **Co-morbidity including number of years on Dialysis** \| \| \| \| \| 0  On dialysis less than 1 year and healthy otherwise \| 1  Dialyzed for 1-4 years, or mild co-morbidity (excluding MCC) \| 2  Dialyzed > 4 years, or moderate co-morbidity  (including MCC) \| 3  Any severe, multiple  Co-morbidity  (2 or more MCC) \| |
| --- | --- | --- | --- | --- | --- | --- | --- | --- | --- | --- | --- | --- | --- | --- | --- | --- | --- | --- | --- | --- | --- | --- | --- | --- | --- | --- | --- | --- | --- | --- | --- | --- |

GI: Gastrointestinal, MCC (Major Comorbid Conditions) include Congestive Heart Failure class III or IV, full-blown AIDS (Acquired Immunodeficiency Syndrome), Severe Coronary artery disease, and moderate to severe Chronic Obstructive Pulmonary Disease, major neurological sequelae & metastatic malignancies or s/p recent chemotherapy.

**SECTION C: Assessment of Nutritional status**

12. Dry weight (Current post dialysis weight) ……….kg

Dry weight (3 months ago---post dialysis weight) ……….kg

1. Height: ………….cm (………..m)
2. BMI …………….kg/m2 BMI=Dry weight/(Height)^2^ kg/m^2^

0 (> 20 kg/m2)

1. (18-19.9 kg/m2)
2. (16-17.99 kg/m2)
3. (< 16 kg/m2)
4. Change in end dialysis dry weight (overall in past 3 months)

0 No decrease in dry weight or weight loss < 0.5 kg

1 Minor weight loss > 0.5 kg but < 1 kg

1. Weight loss > 1 kg but < 5%

3 Weight loss > 5%

1. Decreased fat stores or loss of subcutaneous fat (below eyes, triceps, biceps, chest)
2. Normal (no change)
3. Mild
4. Moderate
5. Severe
6. Signs of muscle wasting (temple, clavicle, scapula, ribs, quadriceps, knee, interosseous)
7. Normal (no change)
8. Mild
9. Moderate

3 Severe
